# Epidemiology and Death-Related Factors of Oncology Patients in Emergency Department 

**Published:** 2016

**Authors:** Bahram Mofid, Kambiz Novin, Elham Sadat Roointan, Mohammad Mehdi Forouzanfar

**Affiliations:** 1 Department of Radiation Oncology, Shohadaye Tajrish Hospital, Shahid Beheshti University of Medical sciences, Tehran, Iran.; 2 Emergency Department, Shohadaye Tajrish Hospital, Shahid Beheshti University of Medical sciences, Tehran, Iran.

**Keywords:** Oncology service, hospital, hospital mortality, epidemiology, emergency medicine

## Abstract

**Introduction:**

Accurate diagnosis and proper treatment of oncology patients presented to emergency department (ED) can dramatically enhance their quality of life and decrease their mortality rate. Therefore, the present study aimed to evaluate these patients from an epidemiologic point of view as well as identifying death-related factors.

**Methods:**

In this retrospective cross-sectional study, all the oncology patients presented to ED during one year were evaluated using census sampling. A checklist that consisted of clinical and demographic data as well as patients’ outcome was filled for each patient. Using SPSS 21, multivariate stepwise logistic regression analysis was done to identify independent death-related factors.

**Results:**

568 patients with the mean age of 53.64 ± 18.99 years were studied (56.5% male). The most common locations of tumor were brain (32.7%) and gastrointestinal tract (27.1%). Pain (32.5%) was the most frequent chief complaint on ED arrival. The overall mortality rate of studied patients was 154 (27.1%), 25 (16.2%) of them in ED. Among the evaluated factors, marital status, visiting on a weekday, arrival to ED via ambulance, type of cancer, stage of cancer, presence of metastasis, being under treatment with chemo-radiotherapy, chief complaint on arrival, tumor location, and admission to intensive care unit (ICU) correlated significantly with in-hospital mortality.

**Conclusion:**

The most common type of cancer in the studied patients was solid, located in the brain or gastrointestinal tract, in stage III and IV, metastatic, and under chemo-radiotherapy. Independent death-related factors included ICU admission, presentation with loss of consciousness or bleeding, arrival via ambulance, cancer stage > II, neuroendocrine and genitourinary location of cancer, and being under chemo-radiotherapy.

## Introduction:

Cancer is the second cause of death behind cardiovascular diseases, worldwide (1). Based on the report of international agency for research on cancer (IARC) in GLOBOCAN 2012, the most common location and highest mortality rate belongs to pulmonary cancer in men and breast cancer in women. Based on the same report, risk of developing cancer before the age of 75 years old is 18.5% for both sexes, while the risk of mortality due to cancer is 10.5% in the same age range. IARC reported the most common cancers in both sexes to be pulmonary, breast, colorectal, prostate, and gastric cancers, in the mentioned order (2). Developing new treatment strategies for cancer patients has led to an increase in their life-span and frequency of emergency department (ED) visits (3). ED is one of the most important places for rapidly addressing the complaints of these patients. Most of these patients visit ED at least once over the course of their disease (4). Recently, many studies have been done to evaluate the different aspects of oncology patients in ED (1, -). Accurate diagnosis and proper treatment of these patients in ED can dramatically enhance their quality of life and decrease their mortality rate (8). Having enough epidemiologic data and a proper plan for managing these patients in ED are necessary for reaching this purpose. Therefore, the present study was designed, aiming to evaluate oncology patients presented to ED from an epidemiologic point of view as well as identifying death-related factors.

## Methods:

***Study design and setting:***

In the present retrospective cross-sectional study, all the oncology patients presented to the ED of Shohadaye Tajrish Hospital, Tehran, Iran, during one year from April 2014 to March 2015, were evaluated using census sampling. No age or sex limitations were implemented in this study. If the patient died on their way to the hospital, they were excluded. The present study was approved by the Ethics Committee of Shahid Beheshti University of Medical Sciences. All the researchers adhered to the principles of Helsinki Declaration during the course of the study.

***Data gathering:***

A checklist that consisted of demographic data (age, sex, marital status, living area, employment), type of arrival to ED, day and time of ED visit, history of visits, patient complaints on arrival, cancer characteristics (type, location, stage, presence and location of metastasis), special treatment characteristics (chemotherapy, radiotherapy, chemo-radiotherapy), and ED disposition and final outcome (discharge from ED, hospitalization in oncology ward or intensive care unit (ICU), mortality) was filled for each patient. The data were extracted from the patients’ clinical profiles. By searching in the medical records unit, all dead, hospitalized, and discharged oncology patients in ED were evaluated.

***Statistical analysis:***

All statistical analyses were done using SPSS version 21. Qualitative variables were reported as frequency and percentage, and quantitative ones as mean and standard deviation. Chi square and Fisher’s tests were used to identify variables that had significant correlation with mortality. In addition, multivariate stepwise logistic regression analysis was done on significant factors to identify independent death-related factors. Type I error (α) was considered 0.05.

## Results:

568 patients with the mean age of 53.64 ± 18.99 years (2 - 94) had visited during the study period (56.5% male). 500 (88%) patients experienced their first visit and 367 (64.7%) were presented in the night shift. 372 (65.5%) patients arrived at the ED in a private car. The most common location of tumor were brain (32.7%) and gastrointestinal (27.1%). 247 (43.5%) of the tumors were metastatic. Tables 1 and 2 depict the baseline characteristics of the patients based on their survival. In addition, table 3 summarizes the final outcome of the patients. The overall mortality rate of studied patients was 154 (27.1%), 25 (16.2%) of them in ED. Among the evaluated factors marital status (p = 0.009), visiting on a weekday (p = 0.044), arrival to ED via ambulance (p < 0.001), type of cancer (p = 0.048), stage of cancer (p < 0.001), presence of metastasis (p < 0.001), being under treatment with chemo-radiotherapy (p < 0.001), chief complaint on arrival (p < 0.001), tumor location (p = 0.04), and hospitalization in ICU (p < 0.001) correlated with in-hospital mortality (tables 1 and 2). Table 4 shows the results of stepwise logistic regression analysis.

**Table 1:** Comparison of baseline characteristics between survived and dead patients

**Variable**	**Total**	**Survival**	**Death**	**P value**
**Sex**				
Female	247 (43.5)	178 (72.1)	69 (27.9)	0.385
Male	321 (56.5)	236 (73.5)	85 (26.5)	
**Age (year)**				
1 - 24.9	39 (6.8)	33 (84.9)	6 (15.4)	0.003
25 - 49.9	173 (30.4)	136 (78.6)	37 (21.4)	
50 - 74.9	271 (47.7)	195 (72)	76 (28)	
75 - 99.9	85 (14.9)	50 (58.8)	35 (41.2)	
**Marital status**				
Single	63 (11.1)	54 (85.7)	9 (14.3)	0.009
Married	505 (88.9)	360 (71.3)	145 (28.7)	
**Employment**				
Employed	316 (55.6)	229 (72.5)	87 (27.5)	0.961
Unemployed	240 (42.3)	176 (73.3)	64 (26.7)	
**Time of arrival**				
Day	200 (35.2)	140 (70)	60 (30)	0.236
Night	367 (64.7)	274 (74.7)	93 (23.3)	
**Day of arrival**				
Weekend	88 (15.5)	57 (64.8)	31 (35.2)	0.044
Weekday	480 (84.5)	357 (74.4)	123 (25.6)	
**Living area**				
Urban	550 (96.8)	399 (72.5)	151 (27.5)	0.235
Rural	18 (3.2)	15 (83.3)	3 (16.7)	
**Transportation to ED**				
Ambulance	182 (32)	70 (38.5)	112 (61.5)	< 0.001
Private car	372 (65.5)	334 (89.8)	38 (10.2)	
**Number of ED visits**				
1	500 (88)	360 (72)	140 (28)	0.333
2	64 (11.3)	50 (78)	14 (21.9)	
3	3 (5)	3 (100)	0 (0)	
**Type of cancer**				
Solid	537 (94.5)	396 (73.7)	141 (26.3)	0.048
Hematologic	31 (5.5)	18 (58.1)	13 (41.9)	
**Stage of cancer**				
I	29 (5.1)	27 (93.1)	2 (6.9)	< 0.001
II	100 (17.6)	96 (96)	4 (4)	
III	128 (22.5)	102 (79.7)	26 (20.3)	
IV	286 (50.4)	167 (58.4)	119 (41.6)	
**Multiple cancers**				
Yes	32 (5.6)	23 (71.9)	9 (28.1)	0.517
No	536 (94.4)	391 (72.9)	145 (27.1)	
**Metastasis**				
Positive	247 (43.5)	147 (59.5)	100 (40.5)	< 0.001
Negative	320 (56.3)	266 (83.1)	54 (16.9)	
**Treatment**				
Chemotherapy	140 (24.6)	95 (67.9)	45 (32.1)	< 0.001
Radiotherapy	32 (5.6)	22 (68.8)	10 (31.3)	
Chemo-radiotherapy	103 (18.1)	59 (57.3)	44 (42.7)	
None	293 (51.6)	238 (81.2)	55 (18.8)	
**ICU admission**				
Yes	20 (3.9)	6 (30)	14 (70)	< 0.001
No	482 (24.1)	371 (77)	111 (23)	

ED: emergency department; ICU: intensive care unit

**Table 2:** Comparison of baseline characteristics between survived and dead patients (continued

**Variable**	**Total**	**Survival**	**Death**	**P value**
**ED chief complaints**				
Fever	12 (2.1)	11 (91.7)	1 (8.3)	< 0.001
Loss of consciousness	132 (23.2)	65 (49.2)	67 (50.8)	
Respiratory distress	51 (8.9)	30 (58.8)	21 (41.2)	
Gastrointestinal disorder	42 (7.3)	35 (83.3)	7 (16.7)	
Pain	185 (32.5)	149 (80.5)	36 (19.5)	
Focal neurologic deficit	91 (16.0)	81 (89)	10 (11)	
Bleeding	16 (2.8)	12 (75)	4 (25)	
Ulcer	13 (2.2)	8 (61.5)	5 (38.5)	
Mass	22 (3.8)	21 (95.5)	1 (4.5)	
Extremity edema	4 (0.7)	2 (50)	2 (50)	
**Tumor location**				
Brain	186 (32.7)	156 (83.9)	30 (16.1)	0.004
Breast	56 (9.9)	40 (71.4)	16 (28.6)	
Prostate	31(5.5)	18 (58.1)	13 (41.9)	
Gastrointestinal	154 (27.1)	106 (68.8)	48 (31.2)	
Respiratory	24 (4.2)	15 (62.5)	9 (37.5)	
Genitourinary	79 (13.9)	51 (64.6)	28 (35.4)	
Lymphoma	4 (0.7)	4 (100)	0 (0)	
Skin	4 (0.7)	3 (75)	1 (25)	
Neuroendocrine	20 (3.5)	15 (75)	5 (25)	
Liposarcoma	1 (0.2)	0 (0)	1 (100)	
Bone	3 (0.5)	3 (100)	0 (0)	
Neck	2 (0.4)	2 (100)	0 (0)	
Heart	1 (0.2)	0 (0)	1 (100)	
Cholangiocarcinoma	2 (0.4)	1 (50)	1 (50)	
Muscle	1 (0.2)	0 (0)	1 (100)	
**Location of metastasis**				
Brain	21 (9)	13 (61.9)	8 (38.1)	0.332
Bone	36 (15.5)	28 (77.8)	8 (22.2)	
Lung	36 (15.5)	22 (61.1)	14 (8.1)	
Multiple	57 (24.5)	24 (42.1)	33 (57.9)	
Pleura	7 (3.0)	5 (71.4)	2 (28.6)	
Uterus	3 (1.2)	3 (100)	0 (0)	
Bladder	4 (1.7)	2 (50)	2 (50)	
Liver	47 (20.2)	28 (59.6)	19 (40.4)	
Pancreas	3 (1.2)	2 (66.7)	1 (33.3)	
Kidney	4 (1.7)	2 (50)	2 (50)	
Rectum	1 (0.4)	0 (0)	1 (100)	
Peritoneum	3 (1.2)	2 (66.7)	1 (33.3)	
Colon	3 (1.2)	2 (66.7)	1 (33.3)	
Pelvic organs	1 (0.4)	1 (100)	0 (0)	
Stomach	1 (0.4)	1 (50)	1 (50)	
Abdominal	2 (0.8)	1 (50)	1 (50)	
Neck	2 (0.8)	2 (100)	0 (0)	

**Table 3:** Outcome of the studied patients

**Outcome**	**Number (%)**
**Emergency ward**	
Discharge	30 (5.3)
Death	25 (4.4)
Hospitalization	513 (90.3)
**Oncology ward**	
Discharge	384 (74.9)
Death	129 (25.1)

**Table 4:** The results of multivariate stepwise logistic regression analysis

**Variables**	**Odds ratio (95% CI)**	**P value**
**ICU admission**		
Yes	4.90 (1.36 - 160.61)	0.027
**Chief complaint**		
Loss of consciousness	3.01 (1.66 - 5.44)	< 0.001
Bleeding	5.20 (0.98 - 27.60)	0.052
**Transportation to ED**		
Private car	0.09 (0.5 - 0.17)	< 0.001
**Stage of cancer**		
II	0.17 (0.05 - 0.53)	0.002
**Treatment**		
Chemo-radiotherapy	2.16 (1.15 - 4.04)	0.016
**Tumor location**		
Neuroendocrine	4.46 (1.05 - 18.94)	0.043
Genitourinary	3.85 (1.78 - 8.29)	0.001

CI: confidence interval; ICU: intensive care unit.

## Discussion:

Based on the results, the most common type of cancer in the studied patients was solid (94.5%), located in the brain (32.7%) or gastrointestinal tract (27.1%), in stage IV (50.4%), metastatic (43.5%), and under treatment with chemo-radiotherapy (49.9%). Finally, 154 (27.1%) patients had died (16.2% in ED) and more than 90% of those who had visited ED had needed hospitalization in the oncology ward. The independent death-related factors were hospitalization in ICU, ED presentation with loss of consciousness or bleeding, arrival via ambulance, cancer stage > II, neuroendocrine and genitourinary location of cancer, and being under chemo-radiotherapy.

Currently, despite the advances in cancer treatment, it is still a major health problem and cancer patients commonly face medical emergencies and unexpected life-threatening diseases (3, 12). These patients are most commonly admitted to ED for decreasing the cancer-related symptoms, controlling treatment side effects, oncology emergencies, simultaneous diseases, or palliative care (7, 13, 14).

Mean age of oncology patients visiting the ED has been estimated to be between 60 to 68 years in various studies (1, 11, 15, 16).In the present study, mean age of patients was 53.64 ± 18.99 years (2 - 94) and most were in the 50 – 75 age range. 

Regarding sex distribution, the findings of the present study were in line with previous studies (4, 7, 8). 

Epigastric pain, nausea and vomiting, and shortness of breath are among the frequent reported causes of ED visit in previous studies (7, 8, 11). While, in the present study, the most common chief complaint of the patients on ED admission was pain, which is in line with the findings of Kraft Rovere et al., Mayer et al., and Barbera et al. (7, 9, 10, 15). 

In the present study, most cancers were solid (73.7%), which is similar to the Bozdemir et al. study result (88%) (11).

The most common location of tumor in our study was brain (32.7%), followed by gastrointestinal tract (27.1%). The most frequent reported tumor locations are lung, gastrointestinal, and respiratory tracts in similar studies (1, 4, 7, -). Out of the 568 cancer patients presented to the ED, 90.3 % were subsequently hospitalized in the oncology department, 5.3% were discharged, and 4.4% died. Death rate in the ED was estimated to be 8-9% in various studies (4, 8). Lower ED mortality rate (4.4%) in the present study might be due to rapid disposition of the patients to other wards and their higher rate of hospitalization. In other words, ED mortality rate has decreased in return to a rise in other wards’ mortality rate. Based on the findings of the present study, independent death-related factors in this study included hospitalization in ICU, visiting due to loss of consciousness or bleeding, arrival via ambulance, higher stage of cancer, tumor type, and being under chemo-radiotherapy. As can be seen, most of these factors are related to severity of disease on admission. For instance, in the studied ED, most of the patients who had arrived via an ambulance were in a worse condition compared to those who had arrived by themselves or accompanied by relatives, and therefore died more. On the other hand, patients in a more severe condition were more commonly admitted to ICU and naturally had a higher death rate.

It seems that patients who visited the studied ED were similar to the participants of other studies from an epidemiologic point of view and the differences present are a result of the natural differences in hospitals regarding patient admission policies and available specialties. Multi-centric studies can be helpful in this respect. We should be cautious about using the results of this study since the study design has some limitations for this kind of conclusion.

## Conclusion:

Based on the results, the most common type of cancer in the studied patients was solid, located in the brain or gastrointestinal tract, in stage III and IV, metastatic, and under chemo-radiotherapy. The factors correlating with hospital mortality included hospitalization in ICU, ED presentation with loss of consciousness or bleeding, arrival via ambulance, cancer stage > II, neuroendocrine and genitourinary location of cancer, and being under chemo-radiotherapy.
